# Directed evolution of a soluble human DR3 receptor for the inhibition of TL1A induced cytokine secretion

**DOI:** 10.1371/journal.pone.0173460

**Published:** 2017-03-09

**Authors:** Itay Levin, Marianna Zaretsky, Amir Aharoni

**Affiliations:** 1 The National Institute for Biotechnology in the Negev (NIBN), Ben-Gurion University of the Negev, Be’er Sheva, Israel; 2 Department of Life Sciences, Ben-Gurion University of the Negev, Be’er Sheva, Israel; Institut National de la Santeet de la Recherche Medicale (INSERM), FRANCE

## Abstract

TNF-like 1A (TL1A) is a cytokine belonging to the TNF superfamily that promotes inflammation in autoimmune diseases. Inhibiting the interaction of TL1A with the endogenous death-domain receptor 3 (DR3) offers a therapeutic approach for treating TL1A-induced autoimmune diseases. Here, we generated improved DR3 variants showing increased TL1A binding affinity and stability using a directed evolution approach. Given the high cysteine content and post-translational modification of DR3, we employed yeast surface display and expression in mammalian cell lines for screening, expression and characterization of improved DR3 variants. A cell-based assay performed with the human TF-1 cell line and CD4^+^ T cells showed that two improved DR3 mutants efficiently inhibited TL1A-induced cell death and secretion of IFN-γ, respectively. These DR3 mutants can be used as drug candidates for the treatment of inflammatory bowel diseases and for other autoimmune diseases, including rheumatic arthritis and asthma.

## Introduction

The mammalian immune system is a complex network of cells regulated by signals transmitted by many secreted and receptor proteins. These include the human tumor necrosis factor (TNF) superfamily that comprises at least 19 members and represents a major class of stimulatory proteins. TNF-like 1A (TL1A) is a newly described member of the TNF superfamily that was shown to be involved in a range of autoimmune inflammatory diseases, including inflammatory bowel diseases (IBD), rheumatic arthritis (RA), and asthma [1–3]. TL1A is currently the only known ligand for death-domain receptor 3 (DR3), which is predominantly expressed by activated T cells and endothelial cells [4,5]. Binding of TL1A to DR3 triggers proliferative signals, most likely via the activation of NF-κB-associated pathways [4]. It was shown that TL1A increases IFN-γ production by acting in synergy with IL-12 and IL-18 and can thus bias the immune response towards a type 1 T helper cell (T_H_1)-like response [6,7]. It was first shown that TL1A is expressed by endothelial cells, with such expression being significantly enhanced by treatment with TNF-α or IL-1 [8]. Subsequent studies have shown that TL1A is also expressed by lymphocytes plasma cells and monocytes, especially in the intestinal tissues of patients suffering from inflammatory bowel diseases (IBD) [1,6].

DR3, the receptor for TL1A, is expressed by CD4^+^ T cells and natural killer cells, with such expression being increased upon T cell activation [1,7]. Although DR3 possesses an intracellular death domain that can used to initiate apoptosis, functional data suggest that the activity of DR3 is mainly pro-inflammatory [2,6,9]. Interestingly, another natural receptor for TL1A is the Decoy Receptor 3 (DcR3), corresponding to a soluble protein that can bind TL1A with high affinity [10,11]. This soluble receptor exhibits broad specificity and can bind other TNF ligands, including FasL and LIGHT [10,12]. Thus, it is difficult to define the net contribution of DcR3 to host immunity due to the diverse functions of the three TNF ligands.

Using two distinct animal models for Chron's disease (CD), it was shown that the induction of intestinal inflammation is associated with significant up-regulation of TL1A and DR3 in the inflamed mucosa [4,13]. Subsequent study using DR3-deficient mice showed that DR3 expression on T cells is required for immunopathology, including the local T cell accumulation and cytokine production seen in experimental autoimmune encephalomyelitis (EAE) and allergic lung inflammation disease models [3,9,14]. Immunopathology and clinical disease were dramatically reduced in DR3-deficient mice, both in a mouse model of lung inflammation and in EAE [9]. In addition, it was shown that genetic variations in the *TL1A* gene contribute to susceptibility to IBD in Japanese and European populations [15]. Finally, several studies using experimental models for RA have shown that the TL1A-DR3 interaction is critical for the pathogenesis of this disease [2,6,16].

The importance of the TL1A pathway in promoting inflammation suggests that blocking TL1A-DR3 interaction could lead to the abolishment of downstream signaling effects and thus prevent various inflammatory disorders. At the same time, the use of antibodies or soluble DR3 receptor able to bind TL1A with high affinity could lead to efficient blocking of TL1A binding to the endogenous DR3 receptor. The advantages of utilizing soluble DR3 receptor are the small molecular weight of the DR3 extracellular domain (ECD) and its perfect recognition of the TL1A binding surface. Moreover, the engineering of DR3 ECD for increased affinity and stability would enhance its ability to compete with the endogenous DR3 receptor for TL1A binding and increase its serum half-life following administration. Indeed, we have previously utilized directed evolution to engineer soluble IL-17A receptor for efficient blocking of IL-17A-mediated inflammatory responses. Our engineered IL-17 receptor exhibits high biological activity and effectively inhibits psoriasis plaque formation in a humanized mice model [17].

Over the past two decades, a directed evolution approach has been extensively used for improving the function of various proteins. This approach is based on the generation of mutants libraries followed by effective screening for the isolation of mutants with improved functions [18,19]. Directed evolution was utilized for increasing the catalytic activity of enzymes and modifying their substrate specificity, for enhancing protein stability and for allowing high soluble expression in recombinant systems. In addition, this approach was used to engineer proteins with extremely high affinity for different ligands [20,21].

**Fig 1:**
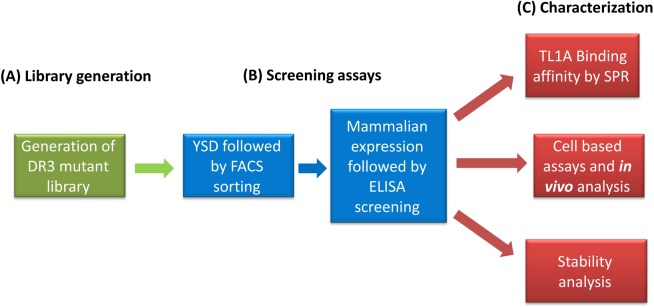
**The directed evolution process for the generation of DR3 mutants with improved TL1A affinity, stability and biological activity comprises: (A) library generation, (B) screening, and (C) characterization**.

In the present work, we utilized directed evolution to generate soluble DR3 ECD variants that show improved stability, augmented TL1A binding affinity and potent inhibition of TL1A-induced cytokine secretion from T cells or apoptosis in a TF-1 cell line. As a first step, we generated a DR3 mutant library containing diversity based on DR3 sequence alignment. We used yeast surface display (YSD) followed by screening of single DR3 mutants expressed in mammalian cells to isolate two improved DR3 variants (Fig 1). These variants exhibited higher affinity, stability and inhibition of TL1A-induced IFN-γ secretion from CD4^+^ cells and apoptosis in TF-1 cells, relative to the native receptor. These results suggest that the engineered soluble DR3 variants can serve as drug candidates for IBD.

## Materials and methods

### Plasmids and cell culture

We amplified the human DR3 extracellular domain from an EST cDNA clone of human DR3 (Open Biosystems). The *E*. *coli* Clooni strain (Lucigen) was used for cloning and plasmid extraction. For the display of DR3 on the yeast cell surface, the DR3 variants were cloned into the pCTCON plasmid using the *Nhe*I and *Bam*HI sites, as previously described [17,22]. For mammalian cell expression, the pFUSE (Invivogen) vector was used to yield DR3-ECD fused with human IgG1 Fc. HEK293T cells were grown in DMEM supplemented with 10% FBS, 2 mM glutamine, 1 x Pen/step solution (all from Biological Industries, Beit-Haemek, Israel). Three hours prior to transfection, the medium was exchanged to Freestyle serum free medium (Invitrogen). H293F cells were grown in Freestyle medium without any supplements. PBL cells and a CD4+ T cell subset were grown at a concentration of 1*10^6^/ml in RPMI 1640, with 10% FBS, 2 mM glutamine, 1x Pen/strep solution supplemented with 10% heat inactivated FBS (all from Biological Industries). TF-1 cells were grown in RPMI medium supplemented with 10% FBS, 2 mM glutamine, 1 x Pen/step solution (all from Biological Industries) and 2 mM GM-CSF (Peprotech). The culture was maintained between 3*10^4^ and 5*10^5^ viable cells/ml. TF-1 cells were obtained from the ATCC, HEK293F cells were obtained from Invitrogen and HEK293T cells were a kind gift from Dr. Ran Taube (Ben-Gurion University, Israel).

### Yeast surface display

The DR3 variants were displayed on the yeast cell surface of EBY100 strain cells and analyzed the cells by flow cytometry, essentially as described [17,22]. For this, yeast cells were transformed with the pCTCON plasmid encoding for DR3 variants. Following transformation, the cells were grown in SDCAA media (20 g sucrose, 6.7 g yeast nitrogen base, 5 g casamino acids, 5.4 g Na_2_HPO_4_ and 8.6 g NaH_2_PO_4_) at 30°C to logarithmic phase and induced as previously described [17]. The cells were subsequently collected, washed with PBSF (PBS + 1 g/L BSA) and labeled with 0.2 μM TL1A (R&D Systems) that was biotinylated using a biotin labeling kit (Pierce) according to manufacturer’s procedure. The cells were then washed and labeled with mouse α-myc antibodies (Santa Cruz Biotechnology, 1 μl/50 μl PBSF). Next, cells were washed and labeled with FITC-conjugated α-mouse IgG (Sigma, 1 μl/50 μl PBSF) and allophycocyanin-conjugated streptavidin (Jackson Immunoresearch, 1 μl/50 μl PBSF). The labeled cells were washed, resuspended with PBSF and analyzed by flow cytometry (FACS Accury, BD) as previously described [17].

### Library generation

A DR3 back-to-consensus library was generated following amplification of the human DR3 gene and digestion of a 5 μg sample with DNaseI to yield 50–125 bp fragments, as previously described [17,23]. The fragments were reassembled, as previously described [17,24], in the presence of a mixture of 16 short oligonucleotides (4–6 nM each). The resulting library contained 3–8 mutations in each gene, with an average of ~4 mutations [23,24]. The reaction mixture was further amplified by nested PCR, as described [17,22]. The libraries were ligated into the pCTCON vector for yeast surface display or cloned by recombination into yeast. The estimated complexity of the library was ~170,000 mutants based on the following equation: N!/(N-K)!*K, where N is the total number of positions (16) and K is the average number of mutations inserted into the gene (4).

### Library selection using yeast surface display

For fluorescence-activated cell sorting (FACS), the naïve DR3 library was induced and labeled with antibodies against c-myc and biotinylated TL1A, as described above. More than 10^7^ of cells displaying the DR3 mutants were labeled and sorted using a FACS (Synergy iCyt). We sorted the top 1–2% of the green and red fluorescence intensity area. These cells (3–5*10^4^) were collected into rich medium and plated for a new round of sorting. The naïve library was sorted using a sorting gate that included the top 5% of fluorescent cells. Three iterative rounds of enrichment were performed.

### Screening of enriched DR3 libraries using ELISA following mammalian cell expression

*DR3* was PCR-amplified from DNA extracted from the last round of FACS sorting as previously described [17], cloned into the pFUSE plasmid and transformed into HEK293T cells. Two to three days post-transfection, the cultured medium containing the secreted DR3 variants were tested for DR3-TL1A interaction using ELISA, as previously described for detection of IL-17 and IL-17RA interactions [17] with minor modifications. ELISA plates (Griener Microlon 96W) were incubated with 0.66 μg/ml monoclonal mouse α-TL1A antibodies (Santa Cruz) for 1 hour and subsequently with 0.6 μg/ml TL1A (Abcam). Next, the plates were incubated with 100 μl of medium from HEK293T cells transfected to express native DR3 or DR3 mutants, harvested 48 or 72 hours post-transfection. As a positive control, DR3-Fc (R&D Systems) was examined at a concentration of 2 μg/ml, while PBS supplemented with 1% BSA served as a negative control. The plates were then incubated with 100 μl of 0.05 μg/ml of biotinylated goat polyclonal α-DR3 antibodies (R&D Systems), followed by incubation with secondary peroxidase-conjugated streptavidin (Jackson, 1:10000 dilution). Reactions were stopped and analyzed as previously described [17].

### Large scale protein expression and purification in mammalian cells

Plasmid pFUSE containing the DR3 variants was transfected into 500 ml of 293F cells in Freestyle medium according to the manufacturer’s procedure. Six days post-transfection, the media were collected and concentrated five-fold using a 10K Amicon ultrafiltration device, the concentrated supernatant was subsequently diluted six-fold with 30 mM Tris-HCl, pH 8.3 (buffer A) and the supernatant was loaded onto a MonoQ column (GE). Next, the column was washed with 20 column volumes of buffer A and the protein was eluted by applying a gradient of 20 column volumes (CVs) of buffer A to buffer B (30 mM Tris-HCl, pH 8.3 and 0.5 M NaCl), with fractions being collected during the entire elution process. Fraction activity was measured by ELISA, and active fractions were pooled dialyzed against PBS containing 1.5 mM DTT and then diluted ten-fold into 50 mM Na-citrate, 25 mM NaCl, pH 5.8 (buffer C). The diluted active fractions were directly loaded on a SP column (GE), and the column was washed with buffer C until the OD 280 was stable. The SP column was eluted by applying a gradient from buffer C to a buffer containing 50 mM Na-citrate, 450 mM NaCl, pH 5.8 (buffer D) with a gradient length of 23 CVs, with fractions being collected during the whole elution process. Samples were run on an SDS PAGE gel and fractions that contained a major band of approximately 60 kDa corresponding to DR3-Fc fusion protein were pooled. The pooled fractions were diluted two-fold into 2 M NH_3_SO_4_, 50 mM Tris-HCl, pH 7.3 and then loaded on a butyl HIC column (GE). The butyl column was washed for more than 20 CVs with 1 M NH_3_SO_4_, 200 mM NaCl, 25 mM Na-citrate, pH 5.8 and eluted by applying a 21 CV gradient to buffer containing 50 mM citrate, 25 mM NaCl, pH 5.8, and elution fractions were collected and analyzed by SDS PAGE. Fractions containing DR3-Fc at a purity of 90% or higher were pooled, dialyzed against PBS containing 1.5 mM DTT and flash-frozen in liquid nitrogen in small aliquots for future use.

### Affinity measurement using Surface Plasmon Resonance (SPR)

To measure the binding affinity of DR3 variants to TL1A, SPR was performed using the ProteOn XPR36 (Bio-Rad) instrument as previously described [17]. All samples were in PBS containing 2 mM DTT. The DR3 variants (5 μg) were diluted in acetate buffer, pH 5.5, and immobilized onto a GLC chip. As a reference, BSA was immobilized onto the chip. The chip was then blocked and TL1A was run at various concentrations (100, 50, 25, 12.5 and 6.25 nM) at 30 μl/min for 300 sec, followed by 10 min period of dissociation. We determined binding parameters with the Langmuir single binding site model, using Bio-Rad ProteOn Manager Software v2.1.2.05.

### Stability in human serum

To examine the stability of the DR3 variants in serum, 20 μg/ml of DR3-FC, H3 and O6 mutants were incubated in human serum type AB (Sigma) at 37 or 42°C. Samples were taken following 30 min of incubation, centrifuged and added to TF-1 cells together with 8 ng/ml TL1A, 10 μg/ml cycloheximide (see detailed description for assaying TF-1 cells, below). Following six hours of incubation, the cells were lysed and cleavage of DEVD-AMC by caspase-3 was monitored in a Tecan Infinite M200 plate reader with the excitation filter set at 350 nm and the emission filter set at 450 nm.

### Thermal denaturation analysis by Circular Dichroism (CD) spectroscopy

To measure thermal denaturation using CD, 120 μg/ml (2.58 μM) of DR3-Fc and mutants in PBS containing 1 mM DTT were denatured by raising the temperature from 30°C to 70°C at a heating rate of 0.37°C/min and a pitch of 0.5°C using a JASCO Peltier PTC 423S apparatus. The CD signal at 215 nm was measured during the denaturation process using a JASCO J815 CD spectrometer. Full spectrum (190–260 nm) measurements were taken at 30°C and 70°C to determine the level of secondary structure in DR3-Fc and mutants. The data were normalized to a fraction of folded protein and the melting temperature (Tm) was derived by fitting the normalized data to a logistic model using SigmaPlot software.

### CD4^+^ cell-based assay for the inhibition of TL1A-induced IFN-γ secretion

PBMCs (peripheral blood mononuclear cell) were isolated from the blood of normal healthy volunteers using Lymphoprep (Axis shield, Oslo, Norway) according to the manufacturer’s instructions. The PBL (Peripheral blood lymphocytes) fraction was isolated by incubation of the PBMCs in complete RPMI medium in a flask at 37°C for three hours, with the non-adherent fraction being designated as the PBL fraction. The isolation of the CD4^+^ T cell subset was performed using a CD4^+^ isolation kit (Miltenyi Biotec, Auburn, CA) as described by the manufacturer. PBL or CD4^+^ cells were incubated with IL-12 (2 ng/ml) and IL-18 (50 ng/ml) with or without TL1A (100 ng/ml) or DR3 variants at different concentrations for 72 h. The cultured media were collected and IFN-γ levels were quantitated using ELISA kits (PeproTech) according to the manufacturer’s procedure.

### TF-1 cell-based assay for the inhibition of TL1A-induced apoptosis

TF-1 cells were seeded at 75,000 cells/well (7.5x10^5^/ml) in RPMI medium containing 1% FBS (Biological Industries) in a black 96-well plate with a clear bottom (Greiner bio-one) at a final volume of 100 μl. Eight ng/ml TL1A, 10 μg/ml cycloheximide, and 0.625–20 μg/ml DR3-Fc (native or mutants) were added, as indicated. After six hours, 100 μl of lysis buffer (50 mM HEPES, 1 mM EDTA, 1% NP-40 detergent, 25 μM DEVD-AMC, pH 7.35) were added to each well and the plate was incubated for ten minutes at 37°C. Subsequently, cleavage of DEVD-AMC by caspase-3 was monitored in a Tecan Infinite M200 plate reader with the excitation filter set at 350 nm and the emission filter set at 450 nm.

### Statistical analysis

All experiments performed with PBL, CD4^+^ and TF-1 cells were repeated at least three times. Data values are presented as means of the three experimental repeats with standard deviation presented as error bars. To examine significance differences between different treatments and different DR3 variant, the data was analyzed by unpaired student t-test. Statistically significance differences were denoted with a star and only p value below 0.05 were considered as statistically significant.

## Results

DR3 is a receptor containing an N-terminal signal peptide followed by an ECD composed of 171 amino acids. The ECD contains 28 cysteines which constitute ~15% percent of the domain and likely form a complex network of disulfide bonds. The large number of cysteines, as well as the two predicated N-glycosylation and three predicated O-glycosylation sites (http://www.cbs.dtu.dk/services/), hinders high expression of this protein in a bacterial system. Previously, our attempts to express a soluble IL-17 receptor led to a high level of aggregated protein that was not active in cell-based assays [17]. Thus, we utilized yeast and mammalian cell lines for DR3 expression to enable screening, isolation and characterization of improved mutants, as described below.

### DR3 library generation

For generation of a DR3 mutant library, we first cloned the full length ECD of the receptor, consisting of 171 residues. We then generated a back-to-consensus DR3 library using sequence alignment of different DR3 ECD orthologues (S1 Fig) [25,26]. Previously, focused gene libraries for directed evolution were generated by integrating structural, functional and evolutionary information available for the protein of interest [27]. Mutagenesis of residues that are different from the consensus sequence of products of the gene family was previously used to generate a back-to-consensus library [25,26]. Screening of these libraries allowed for isolation of mutants with enhanced stability and activity, relative to the native protein [28,29]. To analyze the DR3 ECD sequence and identify residues that are different from the consensus sequence, we aligned sequences of 12 DR3 natural variants. This analysis led to the identification of 13 different residues that deviate from the DR3 consensus sequence (data not shown). We generated a DR3 gene library containing a subset of the 13 target mutations by partially mutagenizing the DR3 gene according to a previously described protocol [30] [23] (S1 Fig). Following library generation, we sequenced six random DR3 variants and found an insertion of 3–8 back-to-consensus mutations per gene with an average of ~4 mutations per gene.

### Enrichment of the DR3 library using yeast surface display

**Fig 2:**
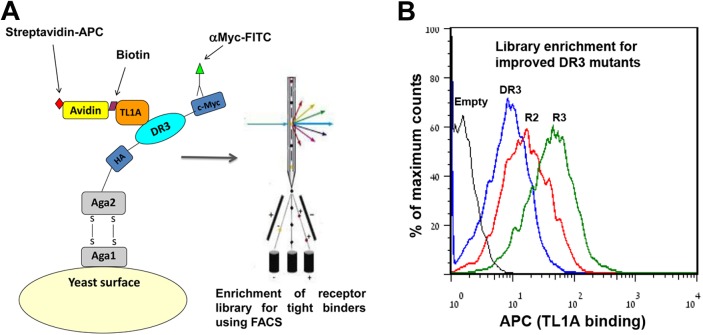
Screening for improved DR3 mutants using yeast surface display. (**A**) Yeast surface display of DR3; DR3 (turquoise) is displayed as an Aga2 (gray) fusion on the surface of the yeast. Expression and TL1A binding are detected by fluorescent antibodies (green) and streptavidin (yellow), respectively. (**B**) Flow cytometry histogram analysis of cell populations displaying native DR3 (blue), and DR3 mutant libraries after two rounds (R2, red) and three rounds of enrichment (R3, green) for TL1A binding. In each round of enrichment, tje cell population exhibiting high expression (FITC signal) and TL1A binding (APC signal) were sorted according to the fluorescent signals. A cell population that was not surface displayed is shown in black. Analysis of cells was performed following incubation with 0.2 μM TL1A.

Previously, yeast surface display proved to be a potent approach for the generation of proteins with improved binding affinity and stability [22]. This approach enables the screening of large mutant libraries using a FACS, allowing for fine tuning of the selection threshold [22]. We utilized this approach to display the DR3 ECD on the yeast cell surface and examine TL1A binding (Fig 2). DR3 display levels were monitored using antibodies labeled with fluorescein isothiocyanate (FITC) and antibodies against the myc tag fused to the DR3 C-terminus. We utilized biotinylated TL1A, followed by labeling with streptavidin-allophycocyanin (APC) to monitor TL1A binding. To screen the naïve library for TL1A binding, we incubated yeast cells displaying the DR3 library with TL1A and analyzed and sorted ~5*10^6^ mutants with a FACS, based on DR3 expression and the binding signal. We performed three iterative rounds of enrichment and observed a continuous increase in the mean fluorescence of the cell population (Fig 2B). However, we found that the selection resulted in accumulation of short truncated sequences of DR3, probably due to non-specific interactions with TL1A (S2 Fig). To overcome this limitation, the library was enriched for full-length DR3 using polyclonal antibodies for the DR3 ECD (see S2 Fig). For cloning of the enriched library into a mammalian expression vector, the third round library plasmids were digested and only the band corresponding to DNA encoding the complete DR3 ECD mutants was sub-cloned into a mammalian expression plasmid for further screening following expression in a mammalian cell line (see below).

### Screening of the enriched DR3 library using ELISA

To enable the identification of single DR3 variants exhibiting improved TL1A binding affinity, we sub-cloned the enriched library from the yeast surface display-based screening into a mammalian vector fused to the sequences encoding an N-terminal leader peptide and a C-terminal human IgG1 Fc. It was previously shown that the extracellular domain of many receptors is heavily glycosylated and that glycosylation can contribute to receptor binding to the target ligand [31]. Thus, the mammalian-based expression of soluble DR3 receptor offers the advantage of subjecting the native protein to post-translational modifications, including glycosylation. To obtain high levels of DR3 expression in mammalian cells, we optimized the leader peptide sequence, as well as transfection and expression conditions in HEK293T cells (see Material and Methods for details). To enable efficient screening of DR3 mutants, we transfected the cells in a 24-well plate format and examined the secreted DR3 for TL1A interaction using ELISA. We developed a fast and sensitive ELISA based on TL1A immobilization, followed by detection of DR3 binding using specific antibodies (S3 Fig). We employed this assay to screen ~250 DR3 mutants expressed in HEK293T cells for binding to TL1A. We identified six mutants exhibiting enhanced binding in supernatant relative to the native protein and further characterized these mutants (S4 Fig).

### Characterization of selected DR3 mutants

To characterize the six selected DR3 mutants, we sequenced the genes and purified the proteins expressed in mammalian cells (see Table 1 for the list of mutations). For mammalian expression, HEK293F cells were transiently transfected and the media were collected seven days post-transfection. We initially attempted to purify the DR3 variants using protein-A affinity chromatography. However, we found a significant loss of DR3 activity following DR3 elution from the protein-A resin due to the extremely low pH of the elution buffer. We thus developed an alternative purification protocol for the DR3 variants that would maintain the specific activity of the proteins. This purification protocol is based on ion exchange chromatography followed by hydrophobic column purification using fast protein liquid chromatography (FPLC; see Material and Methods for details). Following purification, we found that the molecular weight (MW) of the DR3 variants was ~60 kDa, a value that is significantly higher than the 45 kDa calculated molecular mass, based on the primary amino acid sequence (S5A Fig). We further verified that DR3 indeed underwent N-linked glycosylation by subjecting native, H3 and O6 DR3 to Endo-H cleavage, treatment that removes N-linked glycosylation. Upon Endo-H treatment, we observed a decrease of ~10 kDa in the molecular mass of the DR3 variants (S5B Fig), indicating the contribution of N-linked glycosylation to the molecular mass of the protein.

**Table 1:** List of mutations in the six selected DR3 variants.

# Variant	Mutations							# of mutations
A7	E38K	V47P	W56R	A65T	K93A	Q101S		6
I12	H15Q	E34K	V47P	D51G	N61E	Q101S		6
G6	I18T	V47P	D51G	N61E	K93E	Q104P	L129P	7
H3	I18T	V47P	D51G	A65T	K93A	Q101S	L129P	7
O6	I18T	D51G	K93E	L129P				4
N8	H15Q	V47P	N61E	K93E	Q101S	Q104P	L129P	7

**Fig 3:**
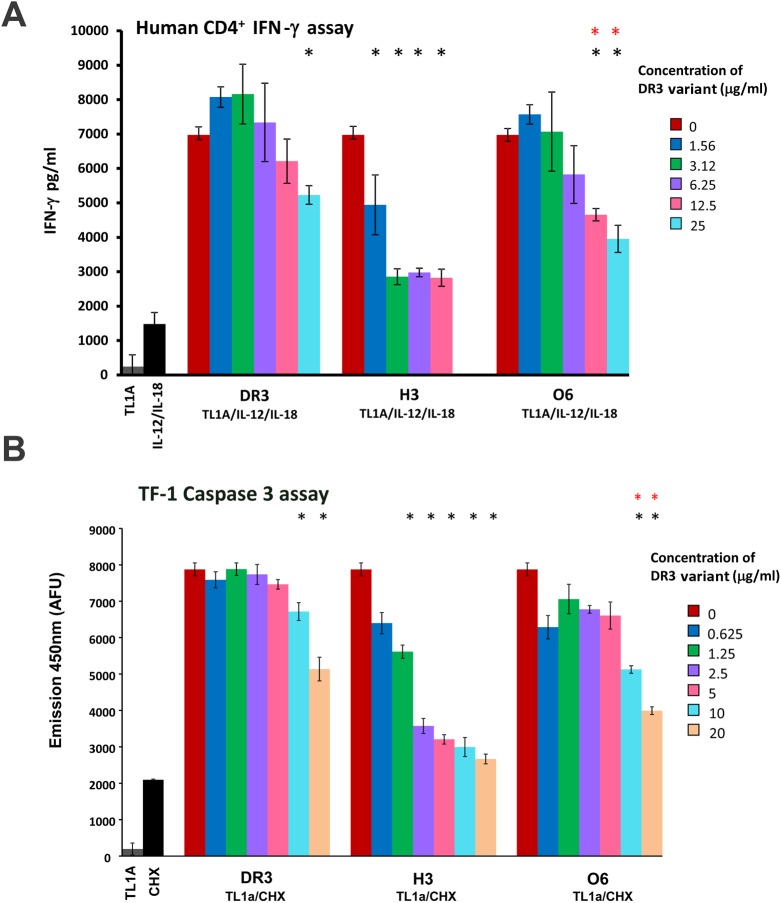
Cell-based assay for the analysis of DR3 variants. (**A**) The H3 and O6 variants are more potent in inhibiting TL1A-induced secretion of IFN-γ in human CD4^+^ than native DR3. Cells were incubated for 72 hours with 100 ng/ml TL1A, 20 ng/ml IL-12 and 50 ng/ml IL-18 and different concentrations of soluble native DR3 or the H3 or O6 receptors. The 1:10 diluted cell supernatant was analyzed by ELISA to assess IFN-γ levels. The IFN-γ values were calculated according to a IFN-γ calibration curve. Black stars denote measurements that are statistically different from no receptor (DR3 = 0) (p<0.02) while red stars are measurements that are statistically different between the O6 and native version of the protein (p<0.05). (**B**) The H3 and O6 mutants are more potent in inhibiting TL1A-induced apoptosis in TF-1 cells than is native DR3. Cells were incubated for six hours with 8 μg/ml cyclohexamide (CHX), 75 ng/ml TL1A and the indicated concentrations of soluble native DR3 or the H3 or O6 receptors. Following six hours of incubation, lysis buffer containing the caspase-3 fluorescent substrate DEVD-AMC was added and caspase-3 activity was monitored for 10 minutes. Black stars denote measurements that are statistically different from no receptor (DR3 = 0) (p< 0.003) while red stars are measurements that are statistically different between the O6 and native versions of the protein (p < 0.05). The data presented in the experiments involving CD4^+^ and TF-1 cells is the average of three independent repeats of each experiment while the error bars represent the standard deviation from the average.

To examine the ability of the engineered DR3 mutants to inhibit TL1A binding to the endogenous DR3 receptor in cells, we established two cell-based assays. The first assay is based on measuring IFN-γ secretion following TL1A addition to human PBL or CD4^+^ cells. Previously, it was shown that TL1A cooperates with IL-12 and IL-18 to induce IFN-γ in PBL and T cells and that the extent of IFN-γ secretion is higher in CD4^+^ cells [7]. Under IBD pathogenic conditions, TL1A induces the secretion of INF-γ from lamina propria mononuclear cells (LPMCs), leading to the generation of proinflammatory responses [4]. Thus, to examine the ability of soluble DR3 variants to inhibit TL1A induced IFN-γ secretion, we added TL1A, IL-12 and IL-18 together with increasing concentrations of soluble DR3 variants to PBL or CD4^+^ cells. The addition of soluble DR3 together with TL1A prevented its binding to the endogenous DR3 receptor due to competition, leading to reduced IFN-γ secretion. We found that the addition of increasing concentrations of soluble DR3 to PBL or CD4^+^ T cells together with TL1A/IL-12/IL-18 led to a gradual decrease in TL1A-induced IFN-γ secretion (Fig 3A). Next, we tested the ability of the different DR3 variants to inhibit TL1A-induced IFN-γ secretion in these cells. We found that the H3 and O6 variants were able to inhibit IFN-γ secretion in human CD4^+^ or PBL cells more efficiently than did the native protein, highlighting the improved biological activity of these variants (Fig 3A and S6 Fig). In contrast, we found that other variants, including N8, I12 and A7, exhibited no improved inhibitory activity relative to the native protein (S7 Fig), while G6 was less potent than O6 (S8 Fig).

To further examine the ability of the H3 and O6 variants to inhibit TL1A-induced activity in cells, we used the TF-1 human erythroleukemic cell line. It was previously shown that TL1A synergies with cycloheximide (CHX) to induce caspase-3 release that can lead to apoptosis in TF-1 cells [8]. We exploited this cell-based assay to examine the ability of the soluble DR3 variants to inhibit TL1A-induced caspase-3 release. To monitor the level of caspase-3 activity following treatment with TL1A and CHX, we used the DEVD-AMC fluorescent caspase-3 substrate. We found that the addition of H3, O6 and native DR3 in conjunction with TL1A and CHX led to a gradual decrease in TL1A-induced apoptosis. We found a significantly higher inhibition of TL1A-induced apoptosis for the H3 and O6 variants, relative to the native protein (Fig 3B). Comparing levels of caspase-3 activity indicated that ~50% of inhibition was achieved at concentrations of 20, 1.25 and 10 μg/ml for the native, H3 and O6 versions of the protein, respectively, indicating a 16-fold improvement in biological activity of the H3 variant relative to the native protein. These results are in good correlation with the PBL/CD4^+^ results, highlighting the ability of the engineered soluble DR3 receptors to inhibit TL1A-induced cellular responses. Next, to examine whether the increases in biological activity of the H3 and O6 variants was correlated to increased TL1A binding affinity, relative to the native protein, we conducted SPR analysis. We found that the H3 and O6 variants exhibited 5- and 4.6-fold increased TL1A binding affinity, relative to native DR3, respectively (Table 2). Such degrees of improvements in TL1A binding affinity are in line with previously reported improvements in the activity of soluble IL-17R isolated from back-to-consensuses library screening [17].

**Table 2:** Kinetic rate constants of TL1A cytokine binding to native DR3-Fc (WT), H3 and O6 variants as determined by SPR.

Immobilized Ligand	*k*_*a*_(1/Ms)*	*k*_*off*_ (1/s)*	K_d_ (M)*	Fold increase in Affinity
WT	1.60* 10^4^	7.20*10^−4^	4.49*10^−8^	-
H3	4.26*10^4^	3.51*10^−4^	8.26*10^−9^	5
O6	3.63* 10^4^	3.57*10^−4^	9.83*10^−9^	4.6

*Values were derived from averaging three individual experiments for each DR3 variant. All Chi^2^ values for the data fitting were below 5%.

**Fig 4:**
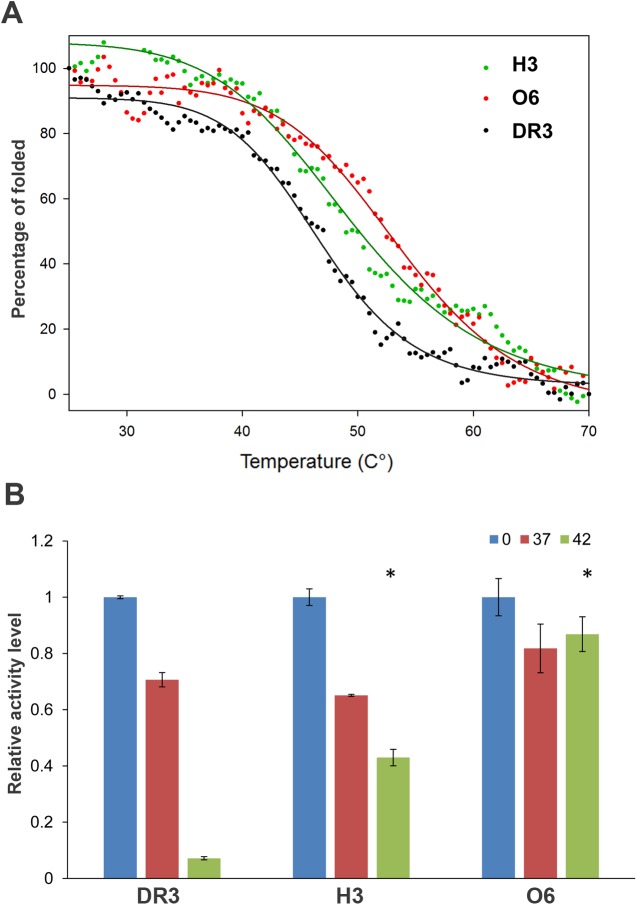
Stability of native DR3, and the H3 and O6 variants. (**A**) Thermal denaturation of DR3 variants measured using CD spectroscopy. The DR3 variants, including the native protein (black), H3 (green) and O6 (red), were subjected to thermal denaturation at 30–70°C at a heating rate of 0.37°C/min, and changes in the ellipticity at 215 nm were monitored. Solid lines represent a logistical model fitting of the melting curves. The O6 and H3 variants exhibit improved melting temperature of 3.5°C and 5.5°C, respectively. (**B**) Stability of native DR3, and the H3 and O6 variants in human serum. The DR3 variants were incubated for 30 minutes in human serum at 0°C, 37°C and 42°C and the residual inhibitory activity of TL1A-induced apoptosis in TF-1 cells was determined (see also Fig 4B). All results were normalized to the DR3 activity following incubation at 0°C. High sensitivity of the native protein relative to mutant activity is observed following incubation at 42°C. Black stars denote measurements that are statistically different than the residual activity of the native protein following incubation at 42°C (p<0.05).

Earlier analysis of engineered proteins containing back-to-consensus mutations indicated a significant increase in the stability of the target protein. We have previously shown that back-to-consensus mutations in selected IL-17R or sulfotransferase 1E1 variants led to significant increases in stability [17,25]. To evaluate the improvement in thermostability of the H3 and O6 DR3 variants, we performed far UV CD spectroscopy. Using this approach, we followed changes in the CD signal at 215 nm upon gradual heating of H3, O6 and native DR3 samples as the temperature rose from 25°C to 70°C (Fig 4A). The melting curves obtained for the different variants indicated an increase of 3.5°C and 5.5°C in the thermostability of the H3 and O6 variants, respectively, relative to the native protein. To further examine the stability of the DR3 variants under more physiological conditions, we incubated the variants in human serum at 37°C and 42°C for 30 minutes and examined their residual ability to inhibit TL1A-induced apoptosis in TF-1 cells (Fig 4B). We found that the H3 and O6 variants exhibited higher residual activity following incubation, relative to the native protein, a finding that is in good correlation with the CD thermostability analysis (Fig 4). Overall, this analysis demonstrated the improved stability of the H3 and O6 variants under different environmental conditions, relative to the native protein.

## Discussion

In this work, we employed directed evolution to generate DR3 variants with improved TL1A affinity, stability and biological activity. Sequence analysis of the six mutants that were initially analyzed showed that some of the mutations were highly abundant (e.g. V47P), while others (e.g. E38K) are found in only one or two variants (Table 1). The H3 variant that exhibited the highest biological activity in two different cell-based assays (Fig 3) contains a unique combination of mutations that confers improved activity, stability and TL1A binding affinity to the protein. Currently, the crystal structure of the ECD of DR3 is unknown and thus it is impossible to map and decipher the structural consequences of the mutations. Nevertheless, to obtain insight into the possible location of the mutations in DR3 structure, we generated a structural model of the ECD using the I-TASSER server (http://zhanglab.ccmb.med.umich.edu/I-TASSER/) [32]. This model is based on the homology of DR3 to the solved TNFR1 receptor structure [33]. The DR3 structural model suggests that the DR3 ECD adopts a rod-like elongated structure, in agreement with the TNFR1 structure [33]. Mapping the mutations found in the H3 and O6 variants on the model structure indicates that the mutations are found on the surface of DR3 at two faces of the molecule (S9 Fig). Further structural analysis of DR3 in complex with TL1A will reveal whether these residues are involved in DR3-TL1A interactions that contribute directly to the affinity between these proteins.

As demonstrated before, the ECD of many receptors is highly glycosylated and such glycosylation can significantly contribute to receptor conformation and binding to the target ligand [31]. Thus, an important component of our screening strategy involved the expression of the DR3 mutants in mammalian cells so as to maintain receptor glycosylation. The combination of yeast surface display screening of large DR3 libraries followed by screening of individual mutants expressed in mammalian cells proved to be an effective approach for developing an engineered human soluble receptor. We have previously utilized this approach for the engineering of improved IL-17R variants with enhanced stability, IL-17A binding affinity and biological activity [17]. In the current directed evolution approach, yeast surface display enabled the screening of over 10^6^ mutants, followed by screening of a manageable size of ~250 individual mutants following expression in mammalian cells. Of the ~250 mutants screened, only two mutants (<1%) exhibited improved activity, suggesting that it would have been extremely difficult to isolate these variants solely based on yeast surface display or HEK293T-based screening systems.

In pathological conditions, binding of TL1A to its native receptor DR3, is associated with several autoimmune diseases, including IBD, asthma, and RA [1–3]. Blocking of TL1A binding to its endogenous DR3 receptor by injection of soluble DR3-Fc may stop downstream signaling effects and thus inhibit these inflammatory disorders. Our cell-based assays in CD4^+^ and TF-1 cells showed the high potency of H3 in inhibiting TL1A-induced IFN-γ secretion and apoptosis, respectively (Fig 3). We observed a ~16-fold increase in potency of the H3 variant relative to the native protein, in correlation with a 5-fold improvement in TL1A binding affinity (Table 2). However, our stability analysis showed that the O6 variant exhibited higher stability than seen with the native protein or the H3 variant, as measured by CD and cell-based assay (Fig 4). Thus, it is hard to predict the *in vivo* efficacy of the two variants. In the future, we will test both variants for *in vivo* stability and efficacy in inhibiting IBD progression in mouse models. Previous work with a TNBS-induced IBD model showed that mice treated with mouse DR3-Fc soluble receptor were much more viable, relative to control mice [34]. Thus, we anticipate that the H3 and O6 variants will serve as highly potent inhibitors of TL1A-induced intestinal inflammation in the TNBS mouse model.

Several soluble receptors are currently used in the clinic as therapeutic drugs, including Rinolacept that binds IL-1 and Etanerecept that binds TNF-α. These soluble receptors exhibit unique therapeutic effects in different individuals and disease states [35]. Soluble trap receptors neutralize the target ligand through their intrinsic ligand-receptor binding pocket, in contrast to antibodies that can bind the target ligand through different interaction regions [36]. The ability to engineer soluble receptors for higher ligand binding affinity and stability may increase the potency and applicability of the latter as therapeutic drugs. In addition, the relatively small size of the DR3 ECD allows its fusion to other inhibitory domains to obtain bi-specific reagents that can simultaneously target two pro-inflammatory agents. Still, there are several developmental steps that must be taken before the DR3 variants described in this work can be offered as viable drug candidates for the treatment of autoimmune diseases. These steps include testing their activity in animal models, obtaining favorable pharmacokinetics and bio-distribution and extensive immunogenicity tests.

## Supporting information

S1 Fig (**A**) Alignment of mammalian DR3 proteins identifies residues that deviate from the family consensus. Highlighted are the V44, D48 and W53 positions of human DR3 that deviate from the family consensus. (**B**) The percentage of identity of each DR3 orthologue to human DR3. (**C**) The oligonucleotide spiking process for obtaining back-to-consensus mutations in the *DR3* gene (see Material and Methods for details).
(TIF)

S2 Fig Yeast surface display of a DR3 library analysis following FACS enrichment. **(A)** Single clones PCR analysis from DR3 library following 1, 3 and 4 rounds of enrichment. A significant enrichment for shorter genes is observed at the 4^th^ round of enrichment impeding subsequent screening using the yeast surface display system. **(B)** Flow cytometry histogram analysis of a cell population displaying a naïve DR3 mutant library (R0- black), and libraries following one round of enrichment (R1-light blue), two rounds of enrichment (R2- orange) and three rounds of enrichment (R3-green), left- assessing the level of DR3 display with anti-DR3 antibodies and right—assessing the display of the full length DR3 using anti-myc antibodies to a myc tag located on the C-terminal of DR3. (**C**) Dot-plot analysis of the TL1A binding analyzed using streptavidin-APC conjugated against biotinylated TL1A and display levels using anti-myc antibodies. The data indicate no significant increase in DR3 display in the third round of enrichment **(D)** Cloning of the full length genes of the FACS-enriched library in the mammalian expression vector. Sub-cloning was performed to avoid contamination of short DR3 variants as false positives (see main text for details).
(TIF)

S3 Fig ELISA experiments for the detection of DR3–TL1A interactions. (**A)** Schematics of the ELISA for DR3 binding to TL1A. The ELISA plate is coated with anti-TL1A antibodies (green) and subsequently, TL1A (blue). Different DR3 variants (red) are then added to the plate and binding to TL1A is detected using specific biotinylated anti-DR3 antibodies as the primary antibody (yellow) and streptavidin-HRP (red). (**B**) DR3 calibration curve. Commercially available native DR3 at five different concentrations was used in the TL1A-binding ELISA assay, as described in Material and Methods.
(TIF)

S4 Fig ELISA TL1A binding signals of the six selected DR3 variants obtained during the screening of the ~250 DR3 variants in mammalian cells (see main text for detailed description). ELISA binding signals are presented as fold increase relative to the ELISA signal obtained with native DR3, used as a control during the screening.
(TIF)

S5 Fig DR3 variants are modified by post-translational modification. (**A**) The molecular weight (MW) of the DR3 variants is ~60 kDa, while the calculated MW is 45 kDa. (**B**) Deglycosylation of native DR3, and the H3 and O6 variants using Endo-H enzyme. Following incubation with the enzyme, a ~10 kDa reduction in the MW of the proteins was observed, indicating the contribution of N-linked glycosylation to the MW of the proteins. The blue error points to the DR3 band on the gel.
(TIF)

S6 Fig The H3 and O6 variants are more potent in inhibiting TL1A-induced secretion of IFN-γ in human PBL cells than is native DR3. Cells were incubated for 72 hours with 100 ng/ml TL1A, 20 ng/ml IL-12 and 50 ng/ml IL-18 and different concentrations of soluble native DR3 and the H3 and O6 variant receptors. The 1:10 diluted cell supernatant was analyzed by ELISA for detection of IFN-γ levels. The IFN-γ levels presented here were calculated according to an IFN-γ calibration curve. Black stars denote measurements that are statistically different from no receptor (DR3 = 0) with a p < 0.03 while red stars are measurements that are statistically different between the O6 and native versions of the protein (p < 0.05).
(TIF)

S7 Fig The N8, I12 and A7 variants show no improvement in inhibiting TL1A-induced secretion of IFN-γ in human PBL cells relative to native DR3. In contrast, the H3 and G6 variants are improved, relative to the native protein (see also **S4 Fig** and **S6 Fig**). Cells were incubated for 72 hours with 100 ng/ml TL1A, 20 ng/ml IL-12 and 50 ng/ml IL-18 and different concentrations of soluble native DR3 and the G6, N8, I12 variants (**A**) or native DR3 and H3 and A7 variant (**B**) receptors. The 1:10 diluted cell supernatant was analyzed by ELISA for detection of IFN-γ levels. The IFN-γ levels presented here were calculated according to an IFN-γ calibration curve. Black stars denote measurements that are statistically different (p < 0.05) from no receptor (DR3 = 0).
(TIF)

S8 Fig The O6 variant exhibits higher inhibition of TL1A-induced secretion of IFN-γ in human PBL cells relative to the G6 and native versions of DR3. Cells were incubated for 72 hours with 100 ng/ml TL1A, 20 ng/ml IL-12 and 50 ng/ml IL-18 and different concentrations of soluble DR3 variants. The 1:10 diluted cell supernatant was analyzed by ELISA for detection of IFN-γ levels. The IFN-γ levels presented here were calculated according to an IFN-γcalibration curve. Black stars denote measurements that are statistically different (p < 0.05) from no receptor (DR3 = 0).
(TIF)

S9 Fig Mapping of the mutations identified in the H3 and O6 variants onto a model structure of DR3. The structural model of DR3 was generated using the I-TASSER server (http://zhanglab.ccmb.med.umich.edu/I-TASSER/). The structure shown was generated using the UCSF Chimera program and is presented from the surface view. The positions mutated in the engineered variants are located on the surface of the DR3 model structure and are located at two faces of the molecules (A- front view and B-back view). The I18, V47, D51, N61, K93, Q101, Q104, L129 (**Table 1**) positions are highlighted in purple.
(TIF)
