# Supplementary figures and images for: Directed evolution of a soluble human DR3 receptor for the inhibition of TL1A induced cytokine secretion
Source: PLoS One. 2017 Mar 9;12(3):e0173460. doi: 10.1371/journal.pone.0173460 (PMC5344418; doi:10.1371/journal.pone.0173460)

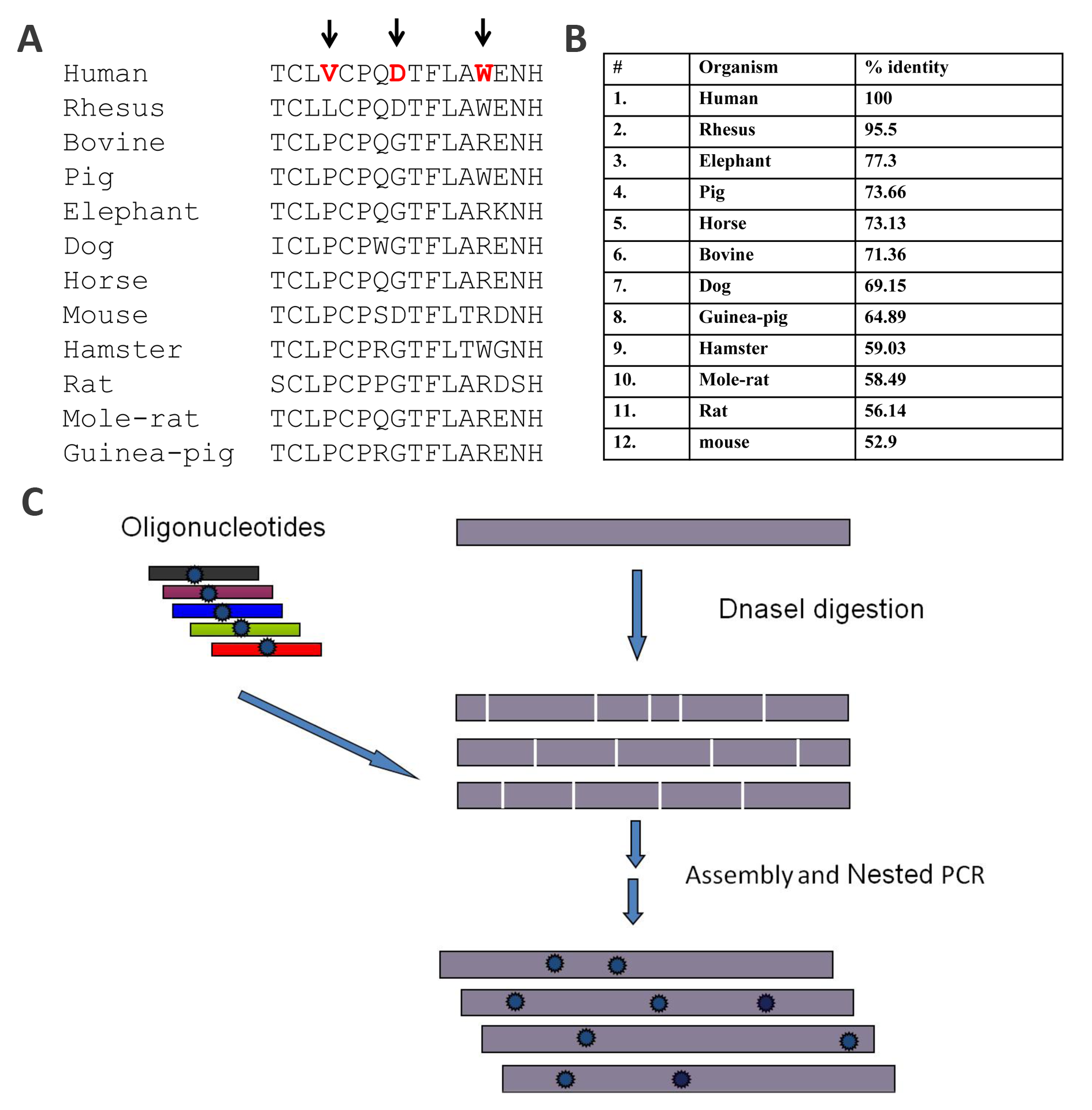

Supplement: S1 Fig — (A) Alignment of mammalian DR3 proteins identifies residues that deviate from the family consensus. Highlighted are the V44, D48 and W53 positions of human DR3 that deviate from the family consensus. (B) The percentage of identity of each DR3 orthologue to human DR3. (C) The oligonucleotide spiking process for obtaining back-to-consensus mutations in the DR3 gene (see Material and Methods for details). (TIF) [file pone.0173460.s001.tif]

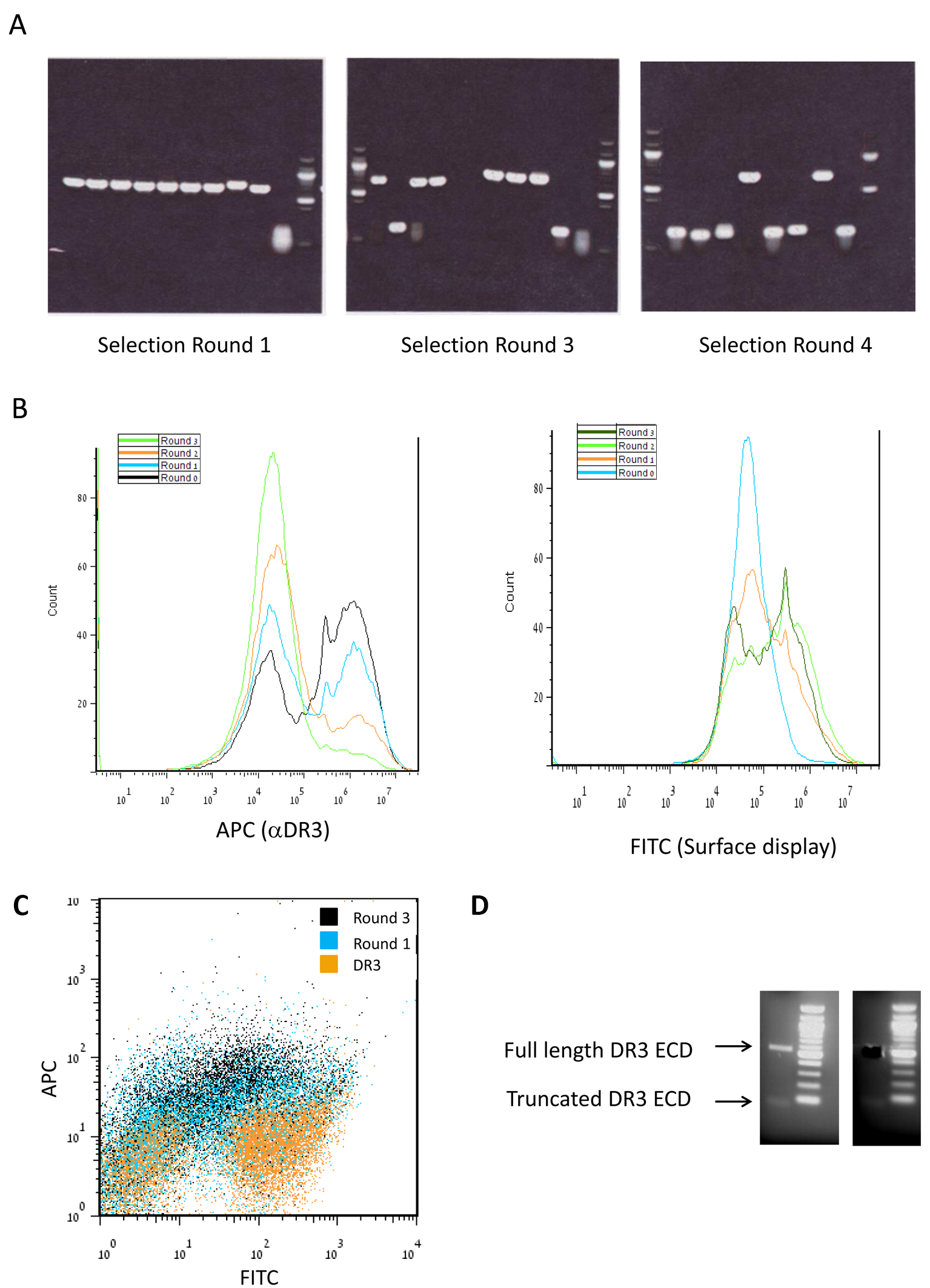

Supplement: S2 Fig — (A) Single clones PCR analysis from DR3 library following 1, 3 and 4 rounds of enrichment. A significant enrichment for shorter genes is observed at the 4th round of enrichment impeding subsequent screening using the yeast surface display system. (B) Flow cytometry histogram analysis of a cell population displaying a naïve DR3 mutant library (R0- black), and libraries following one round of enrichment (R1-light blue), two rounds of enrichment (R2- orange) and three rounds of enrichment (R3-green), left- assessing the level of DR3 display with anti-DR3 antibodies and right—assessing the display of the full length DR3 using anti-myc antibodies to a myc tag located on the C-terminal of DR3. (C) Dot-plot analysis of the TL1A binding analyzed using streptavidin-APC conjugated against biotinylated TL1A and display levels using anti-myc antibodies. The data indicate no significant increase in DR3 display in the third round of enrichment (D) Cloning of the full length genes of the FACS-enriched library in the mammalian expression vector. Sub-cloning was performed to avoid contamination of short DR3 variants as false positives (see main text for details). (TIF) [file pone.0173460.s002.tif]

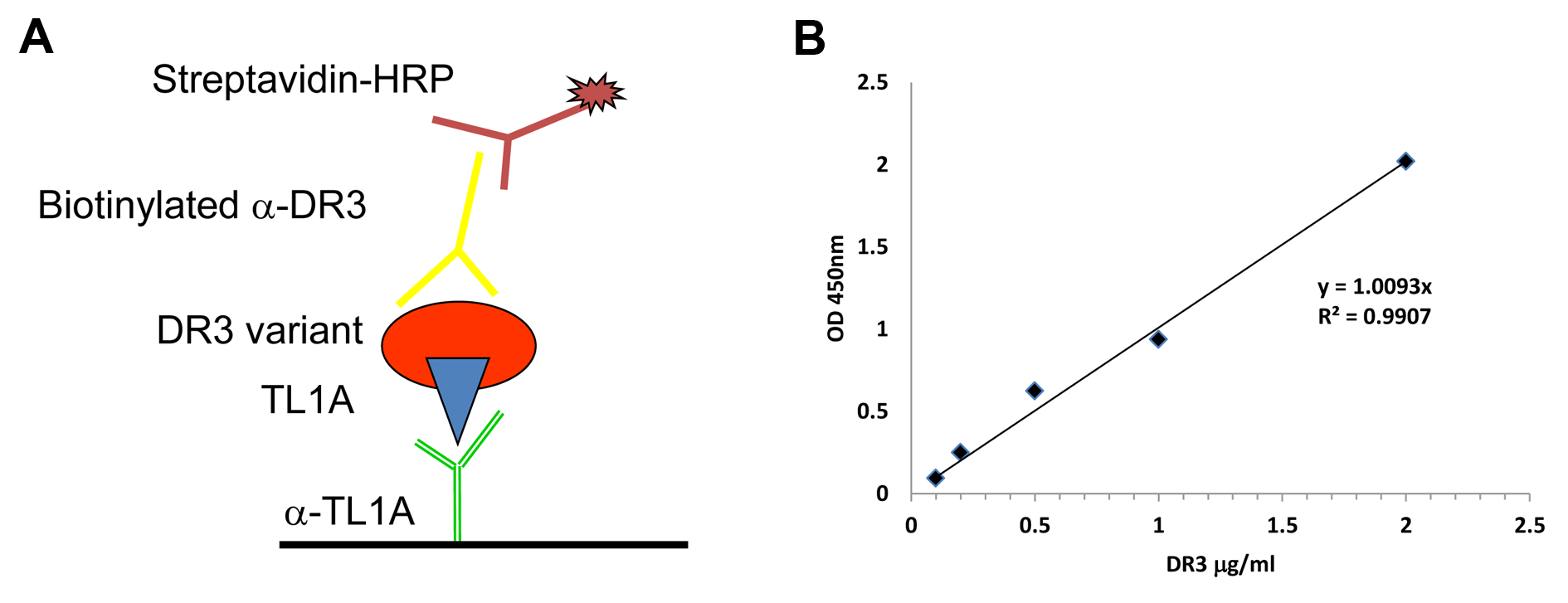

Supplement: S3 Fig — (A) Schematics of the ELISA for DR3 binding to TL1A. The ELISA plate is coated with anti-TL1A antibodies (green) and subsequently, TL1A (blue). Different DR3 variants (red) are then added to the plate and binding to TL1A is detected using specific biotinylated anti-DR3 antibodies as the primary antibody (yellow) and streptavidin-HRP (red). (B) DR3 calibration curve. Commercially available native DR3 at five different concentrations was used in the TL1A-binding ELISA assay, as described in Material and Methods. (TIF) [file pone.0173460.s003.tif]

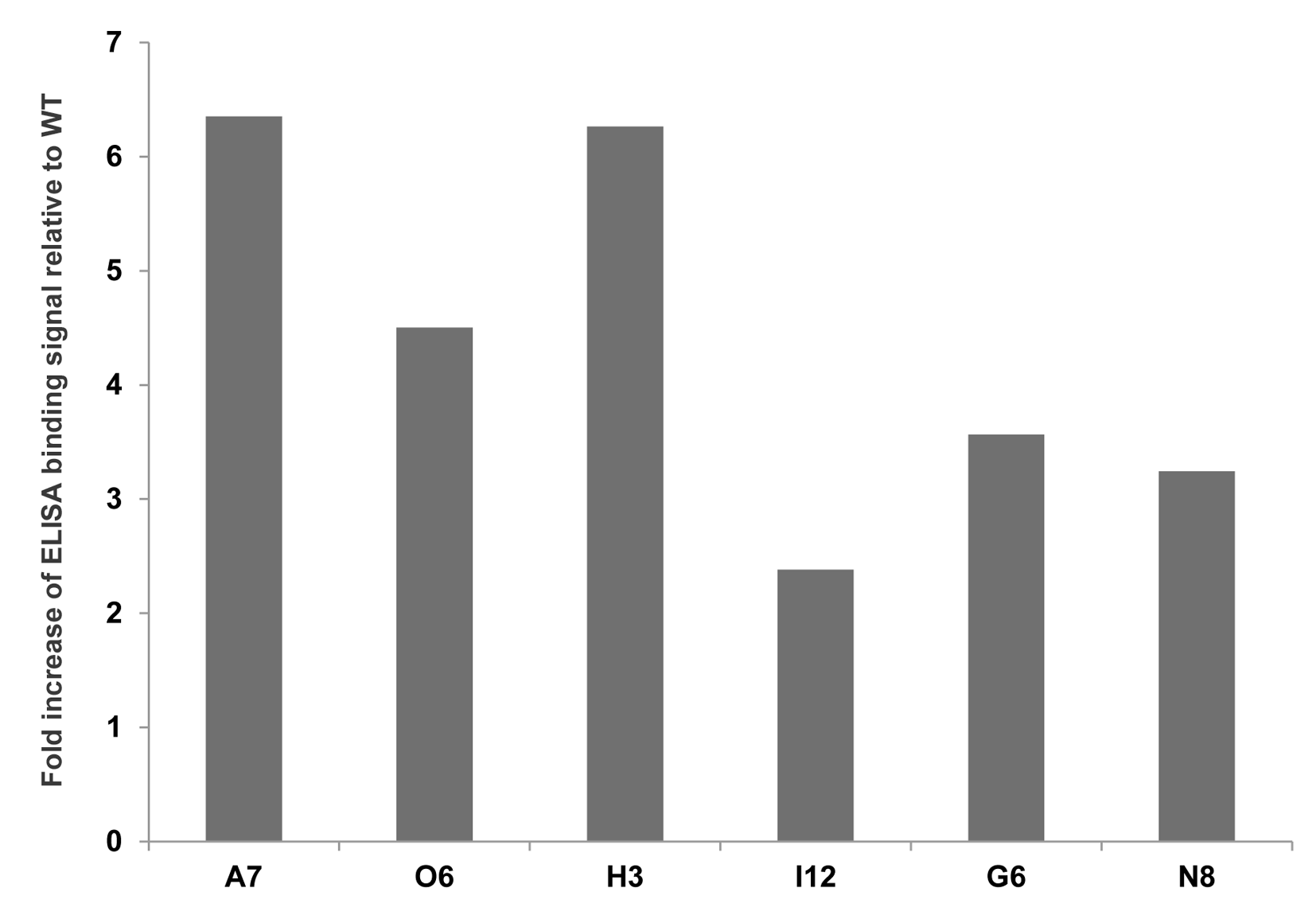

Supplement: S4 Fig — ELISA binding signals are presented as fold increase relative to the ELISA signal obtained with native DR3, used as a control during the screening. (TIF) [file pone.0173460.s004.tif]

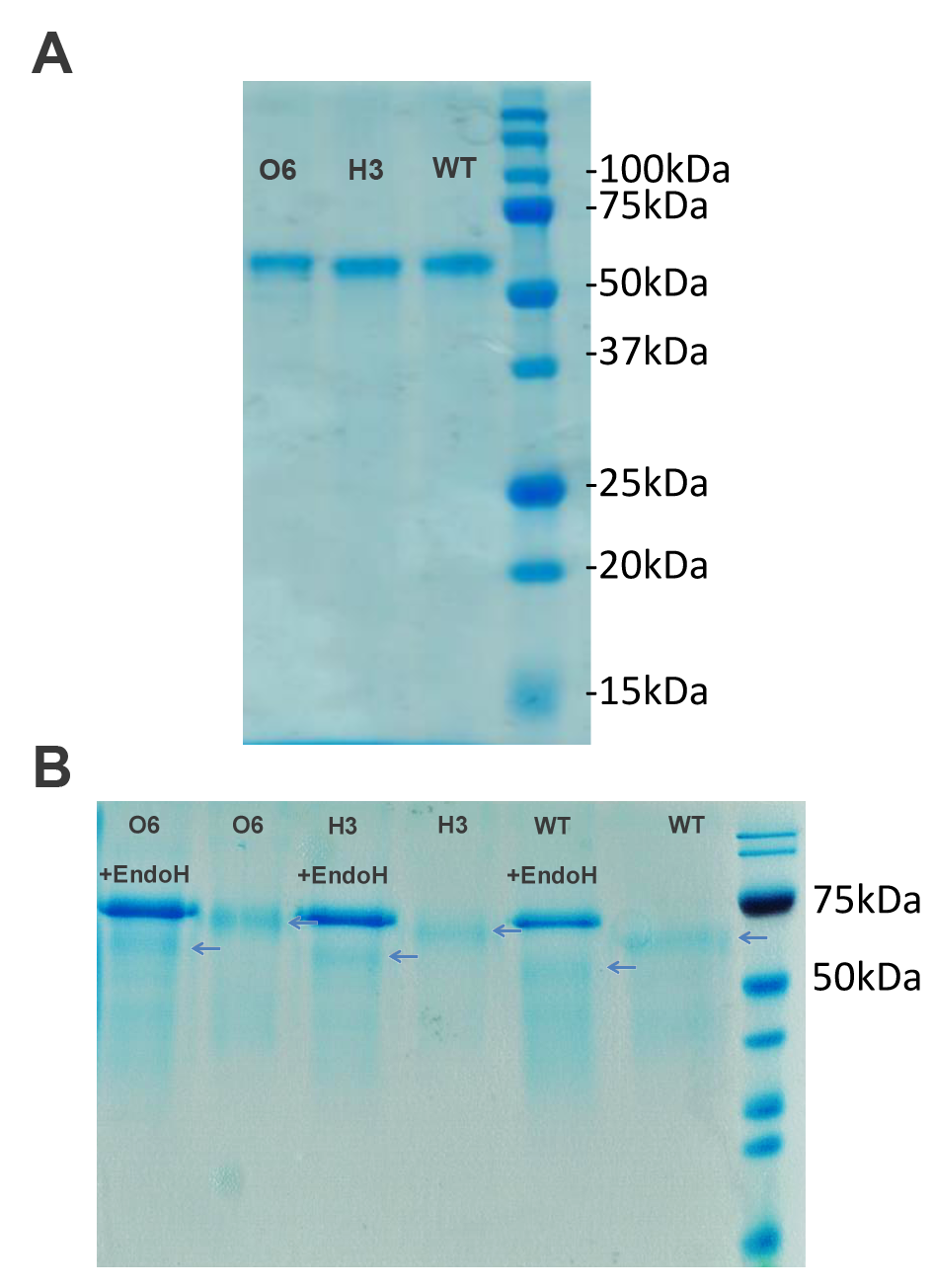

Supplement: S5 Fig — (A) The molecular weight (MW) of the DR3 variants is ~60 kDa, while the calculated MW is 45 kDa. (B) Deglycosylation of native DR3, and the H3 and O6 variants using Endo-H enzyme. Following incubation with the enzyme, a ~10 kDa reduction in the MW of the proteins was observed, indicating the contribution of N-linked glycosylation to the MW of the proteins. The blue error points to the DR3 band on the gel. (TIF) [file pone.0173460.s005.tif]

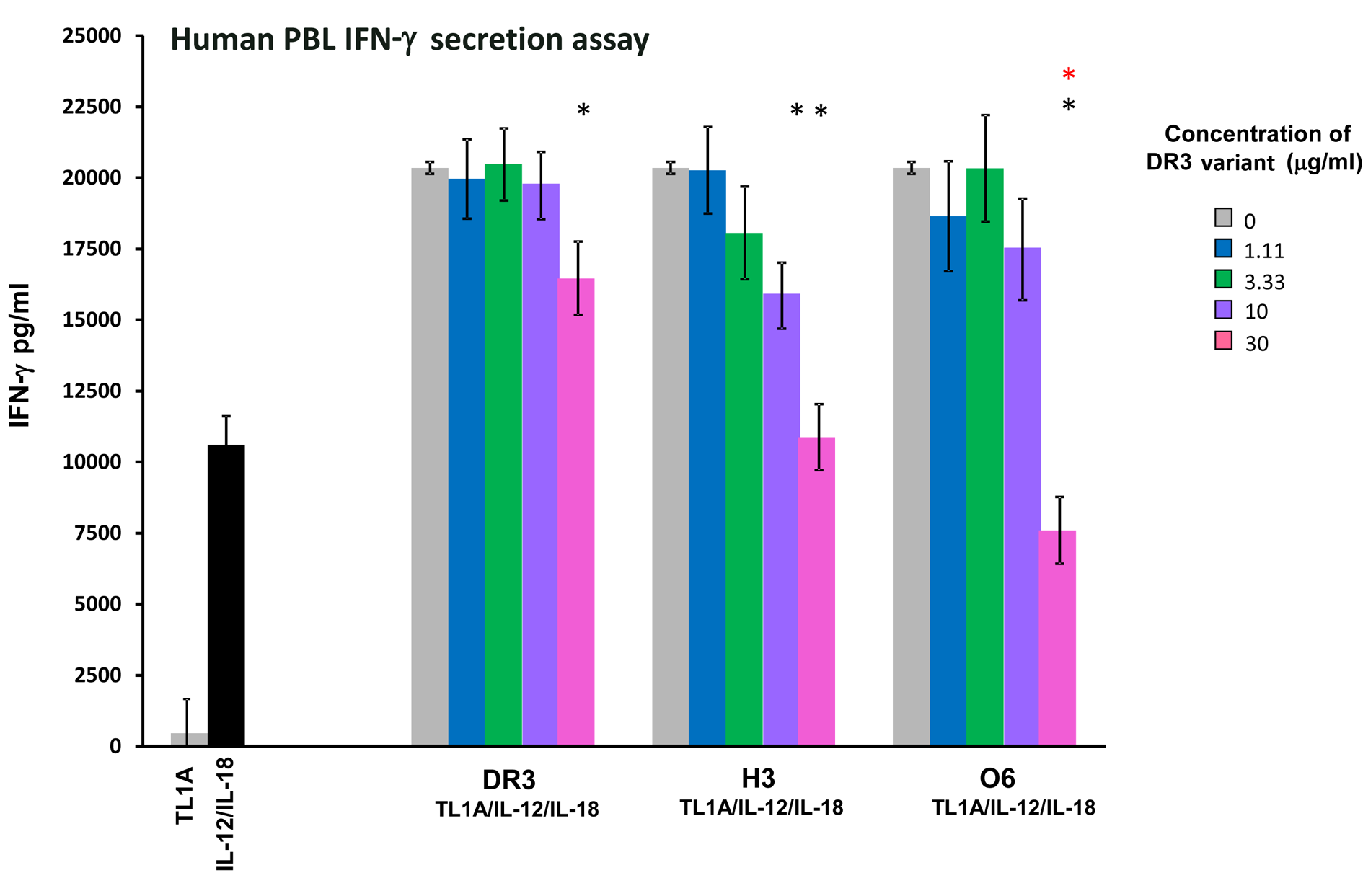

Supplement: S6 Fig — Cells were incubated for 72 hours with 100 ng/ml TL1A, 20 ng/ml IL-12 and 50 ng/ml IL-18 and different concentrations of soluble native DR3 and the H3 and O6 variant receptors. The 1:10 diluted cell supernatant was analyzed by ELISA for detection of IFN-γ levels. The IFN-γ levels presented here were calculated according to an IFN-γ calibration curve. Black stars denote measurements that are statistically different from no receptor (DR3 = 0) with a p < 0.03 while red stars are measurements that are statistically different between the O6 and native versions of the protein (p < 0.05). (TIF) [file pone.0173460.s006.tif]

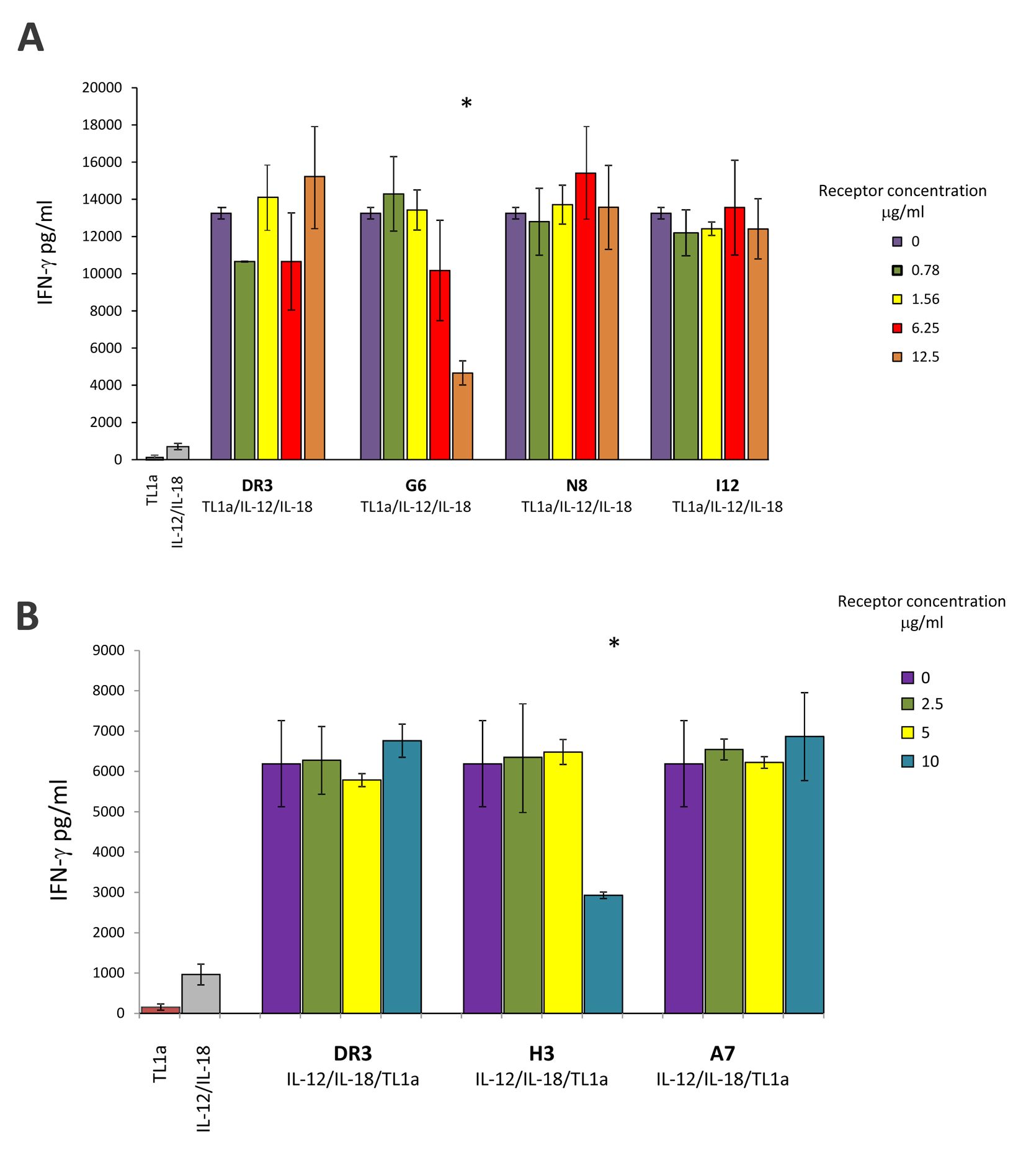

Supplement: S7 Fig — In contrast, the H3 and G6 variants are improved, relative to the native protein (see also S4 Fig and S6 Fig). Cells were incubated for 72 hours with 100 ng/ml TL1A, 20 ng/ml IL-12 and 50 ng/ml IL-18 and different concentrations of soluble native DR3 and the G6, N8, I12 variants (A) or native DR3 and H3 and A7 variant (B) receptors. The 1:10 diluted cell supernatant was analyzed by ELISA for detection of IFN-γ levels. The IFN-γ levels presented here were calculated according to an IFN-γ calibration curve. Black stars denote measurements that are statistically different (p < 0.05) from no receptor (DR3 = 0). (TIF) [file pone.0173460.s007.tif]

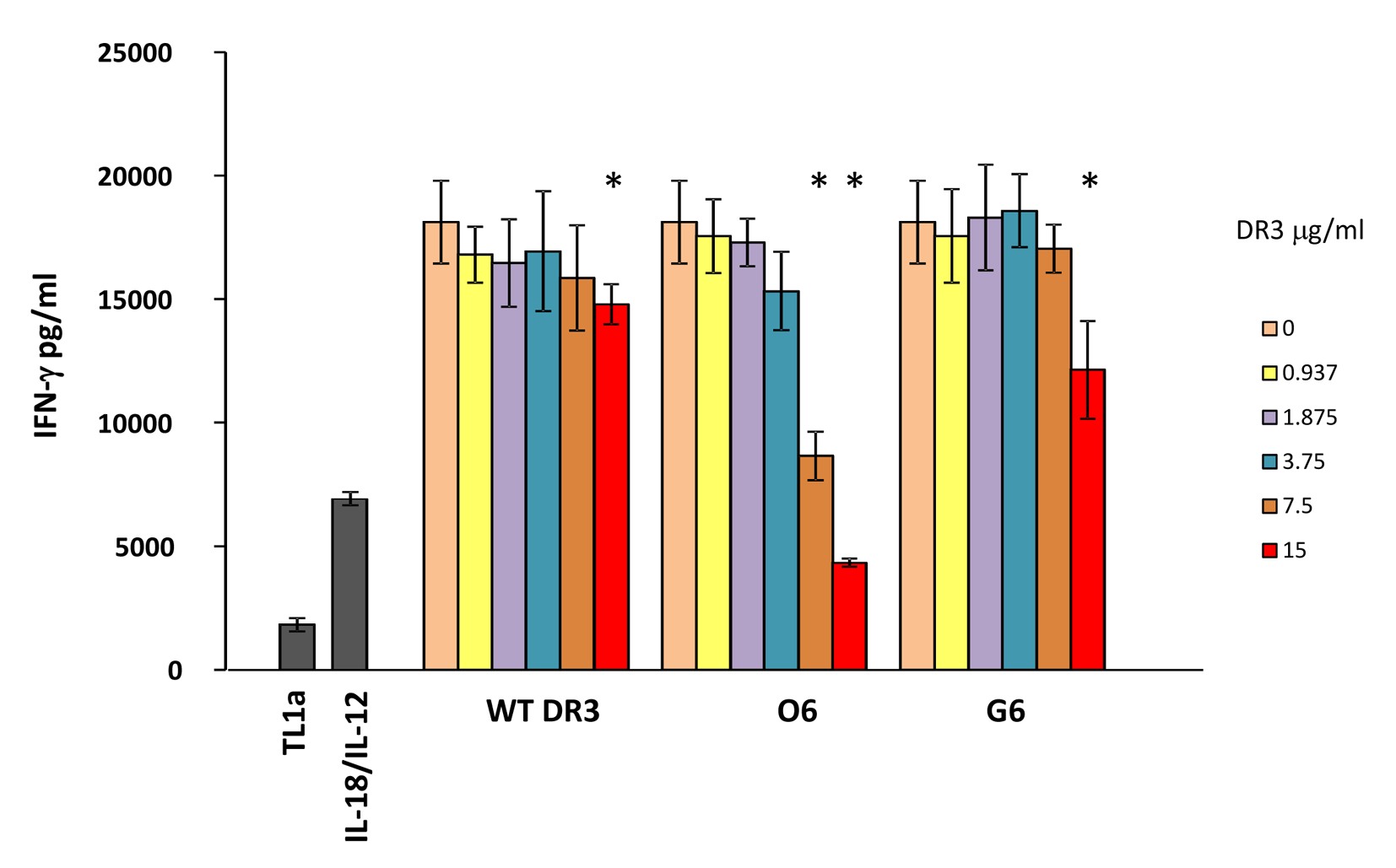

Supplement: S8 Fig — Cells were incubated for 72 hours with 100 ng/ml TL1A, 20 ng/ml IL-12 and 50 ng/ml IL-18 and different concentrations of soluble DR3 variants. The 1:10 diluted cell supernatant was analyzed by ELISA for detection of IFN-γ levels. The IFN-γ levels presented here were calculated according to an IFN-γcalibration curve. Black stars denote measurements that are statistically different (p < 0.05) from no receptor (DR3 = 0). (TIF) [file pone.0173460.s008.tif]

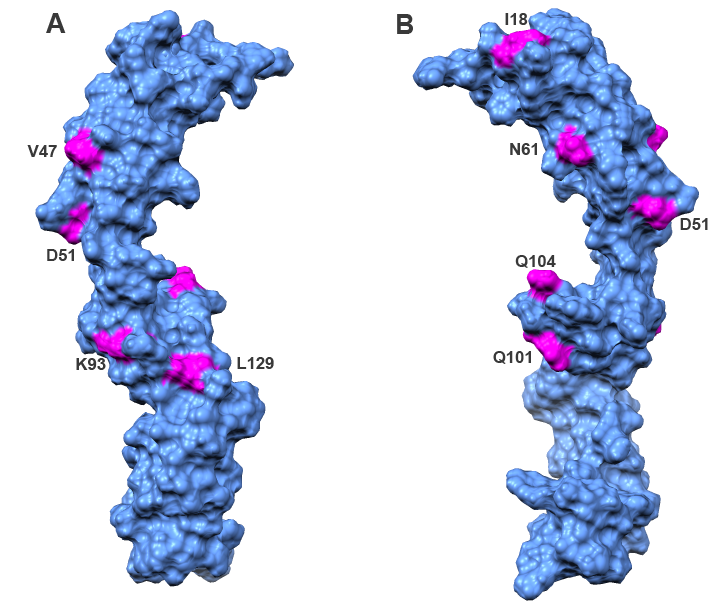

Supplement: S9 Fig — The structural model of DR3 was generated using the I-TASSER server (http://zhanglab.ccmb.med.umich.edu/I-TASSER/). The structure shown was generated using the UCSF Chimera program and is presented from the surface view. The positions mutated in the engineered variants are located on the surface of the DR3 model structure and are located at two faces of the molecules (A- front view and B-back view). The I18, V47, D51, N61, K93, Q101, Q104, L129 (Table 1) positions are highlighted in purple. (TIF) [file pone.0173460.s009.tif]
